# Simultaneous isolation and culture of endothelial colony-forming cells, endothelial cells and vascular smooth muscle cells from human umbilical cords

**DOI:** 10.1007/s11033-025-10418-1

**Published:** 2025-03-13

**Authors:** Marie-Lotus Burger, Steeve Menétrey, Catherine Ponti, Karine Lepigeon, Joanna Sichitiu, Anne-Christine Peyter

**Affiliations:** 1 Neonatal Research Laboratory, Department Woman-Mother-Child, Lausanne University Hospital and University of Lausanne, Lausanne, Switzerland; 2 Clinic of Gynecology and Obstetrics, Department Woman-Mother-Child, Lausanne University Hospital and University of Lausanne, Lausanne, Switzerland; 3 Ultrasound and Fetal Medicine Unit, Department Woman-Mother-Child, Lausanne University Hospital and University of Lausanne, Lausanne, Switzerland

**Keywords:** Human umbilical cord blood, Human umbilical vein, Human umbilical artery, Endothelial cell, Vascular smooth muscle cell, Endothelial colony-forming cell

## Abstract

**Background:**

Regulation of the human umbilical circulation under physiological and pathological conditions remains poorly understood. We previously demonstrated that intrauterine growth restriction (IUGR) is associated with sex-specific alterations in the human umbilical circulation. Our data strongly suggest a differential contribution of subcellular compartmentation depending on fetal sex, vessel type and the presence of IUGR. We therefore developed a protocol to isolate and culture umbilical vascular cells to further investigate the relative contribution of each cell type and subcellular compartmentation to the human umbilical circulation regulation.

**Methods and results:**

Human umbilical cords and cord blood were collected just after delivery. Mononuclear cells were recovered from cord blood using a Ficoll gradient and cultured to obtain endothelial colony-forming cells (ECFCs). Endothelial cells (ECs) were isolated from human umbilical vein (HUV) and arteries (HUAs) by collagenase/dispase digestion, and vascular smooth muscle cells (SMCs) by migration from vascular explants. All cell types were characterized by visualization, and by analysis of biomarkers using immunocytofluorescence and Western blot. ECFCs were also submitted to polychromatic flow cytometry analysis.

**Conclusions:**

This protocol enables simultaneous isolation and culture of ECFCs, HUVECs, HUAECs, HUVSMCs and HUASMCs from the same umbilical cord. It is simpler, faster and more cost-effective than other previously published methods, with good success rates. This will be helpful to further investigate the regulatory mechanisms implicated in the human umbilical circulation under physiological and pathological conditions and to study the influence of fetal sex.

**Supplementary Information:**

The online version contains supplementary material available at 10.1007/s11033-025-10418-1.

## Introduction

Fetal growth and development depend mainly on the functional integrity of the maternal-placental-fetal circulation. In humans, oxygen and nutrients are delivered to the fetus through a single umbilical vein (HUV), while deoxygenated blood containing metabolic waste is returned to the placenta via two umbilical arteries (HUAs).

The regulation of human umbilical circulation under physiological and pathological conditions remains poorly understood. We previously demonstrated that intrauterine growth restriction (IUGR) is associated with sex-specific alterations in the human umbilical circulation [1, 2]. Our data suggested a differential contribution of subcellular compartmentation within vascular smooth muscle cells (VSMCs) depending on fetal sex, vessel type and the presence of IUGR [2].

We therefore aimed to develop a protocol to isolate and culture vascular cells from umbilical cords of healthy or sick neonates to further investigate the relative contribution of each cell type to the human umbilical circulation regulation and assess the influence of biological sex. Indeed, there is growing evidence that this parameter, often neglected in clinical and basic research, plays a key role in physiological and pathophysiological processes, particularly in the cardiovascular system [3, 4].

VSMCs are the predominant cell type in the vascular wall, forming a multicellular layer, whose primary function is to regulate vascular tone. They can contract or relax in response to various stimuli, thereby modulating blood flow and blood pressure.

Endothelial cells (ECs) constitute the monolayer lining the blood vessels’ lumen. They regulate vascular tone by interacting with VSMCs.

Endothelial progenitor cells (EPCs) are circulating components of the endothelium, with vascular repair properties. EPCs can be distinguished according to their phenotype and functional properties [5]: early EPCs appear early in culture, with no capacity to form vessels in vivo, whereas late EPCs or endothelial colony-forming cells (ECFCs) have important proliferative capacity and are able to form a vascular network in vitro and in vivo. Altered circulating EPCs amount and function have been observed in various cardiometabolic disorders [6, 7].

Exploring functional properties of ECs and VSMCs isolated from HUV and HUAs, and of ECFCs from cord blood would contribute to a better understanding of regulatory mechanisms implicated in the human umbilical circulation. Simultaneous isolation and culture of HUVECs, HUAECs, HUVSMCs, HUASMCs and ECFCs from the same patient will not only enable optimal use of each biological sample, but also direct comparison between the different cell types.

During the development of our methodology, we favored the most time- and cost-effective approaches, with limited risk of cross-contamination, while providing satisfactory results when characterizing different cell types using biomarkers.

The present report describes a simple methodology for cell isolation and characterization enabling reliable harvesting of these five vascular cell types from the same umbilical cord.

## Methods

### Umbilical cord collection

The ethical approval was granted by the “Commission cantonale d'éthique de la recherche sur l'être humain (CER-VD)” (protocol number CER-VD-2022-01278). Umbilical cords were obtained from newborns delivered at the Maternity of the University Hospital CHUV in Lausanne, between July 2023 and February 2024. Inclusion criteria encompassed term pregnancies with singleton fetuses; exclusion criteria included neonates with a birth weight above the 90th percentile, fetal abnormalities, genetic syndromes, single HUA, mothers with HIV, hepatitis A, B, or C, and preeclampsia.

**Fig. 1:**
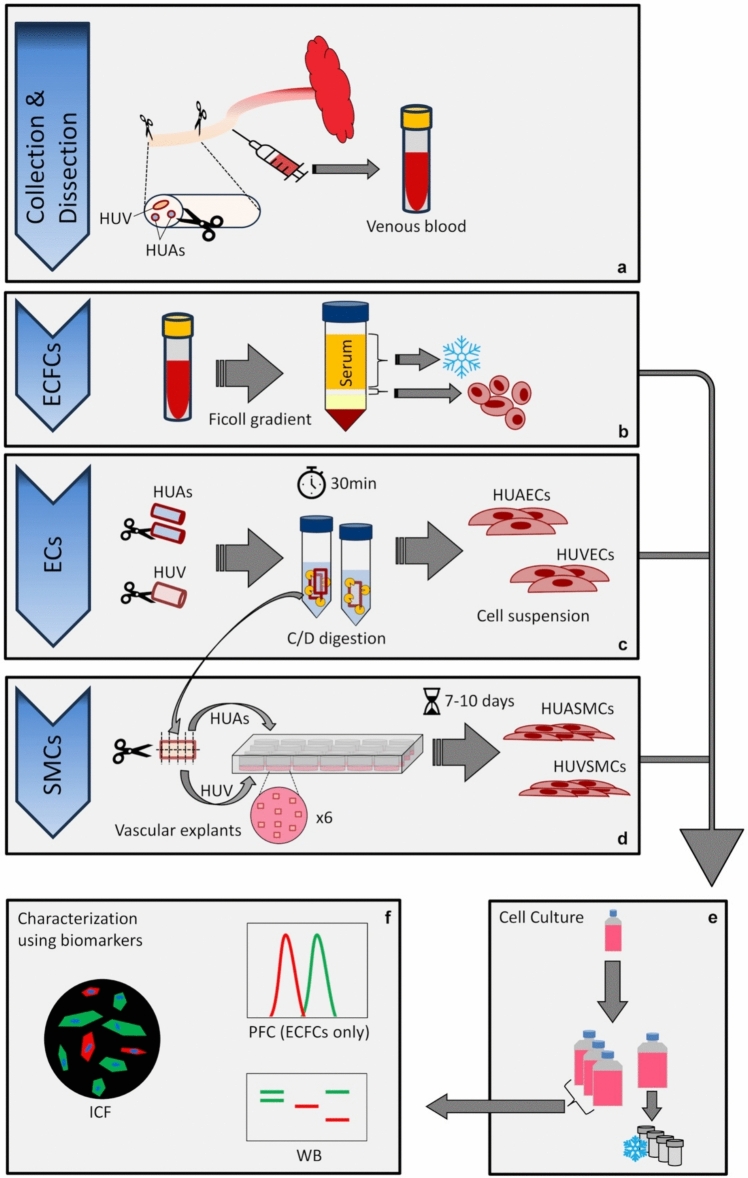
Graphical representation of the main steps to successfully isolate ECFCs, ECs and SMCs from the same human umbilical cord. **a** A 10–15-cm segment of the proximal part of the umbilical cord is harvested just after delivery and dissected to isolate HUAs and HUV. Umbilical blood is punctured from the umbilical vein. **b** ECFCs are isolated from the blood through a Ficoll gradient, and serum is frozen for further investigations. **c** HUAs and HUV are longitudinally opened and incubated in a collagenase/dispase (C/D) solution at 37 °C for 30 min. ECs are harvested from the C/D solution. **d** HUAs and HUV used for EC isolation were washed in PBS and cut into small pieces, distributed into their respective wells, and allowed to grow for 7–10 days. **e** Each cell type is allowed to grow in the appropriate culture medium until subculture in four 75-cm2 flasks. Upon confluency, one flask is used for cryo-preservation, the cells from the other three flasks are used for characterization and further experiments (**f**)

A 10–15-cm segment of umbilical cord was collected as close as possible to the fetus soon after delivery and kept at 4 °C in phosphate-buffered saline (PBS, Gibco, 70013-016) until dissection. Umbilical cord blood was punctured from the HUV in a heparinized tube (S-Monovette® 9ML LH, Sarstedt, 02.1065) and kept at 4 °C until use. The cord blood was used within 12 h and the cord within 24 h after delivery. The methodology described below was validated using 14 umbilical cords to simultaneously isolate ECFCs, HUVECs, HUAECs, HUVSMCs and HUASMCs. Figure 1 summarizes the main steps in the procedure for isolating vascular cells from umbilical cords.

### Cell culture

Each cell type was cultured on plasticwares pre-coated with Type B bovine Gelatin 0.2% (Sigma, G1393-100ML). All culture media were supplemented with 100 µg/ml Primocin® (InvivoGen, ant-pm-05) to prevent contamination by fungi, bacteria and mycoplasma. Unless otherwise stated, culture media were changed twice a week. Cells were trypsinized (PAN-Biotech, PANP10-024100) and subcultured when confluency reached 80–95%. ECFCs and SMCs were frozen in Cryo-SFM (PromoCell, C-29910), and ECs in DMEM containing 20% FBS and 10% DMSO, at a rate of 1C°/min, and stored in liquid nitrogen for further use.

#### ECFC isolation and culture

ECFC isolation was directly inspired by a previously described method [8]. Briefly, cord blood (2-7 ml) was overlaid onto the same volume of Ficoll (Histopaque-1077, Sigma, catalog number 10771) and centrifuged at 740 g for 30 min without brakes. The serum phase (top layer) was harvested and stored at − 80 °C for further investigation. Mononuclear cells were collected from the interphase (white disk) between serum and Ficoll phase. Cells were washed twice with PBS and centrifuged 10 min at 610 g and 430 g before being resuspended in Endothelial Cell Growth Medium MV2 (PromoCell, C-22022). Cells were then transferred to a 25-cm^2^ flask and left for 3 days at 37 °C in a humidified and controlled atmosphere containing 5% CO_2_ before the first medium change. ECFCs were subcultured when confluence reached 80–95% or if some cells in the center of the colonies started to show over-confluence symptoms.

#### EC isolation and culture

The umbilical cord was dissected on ice to isolate 4–6 cm segments from each vessel, which were cleaned from Wharton’s jelly as much as possible, longitudinally opened to expose the lumen and washed in four successive baths of sterile PBS to remove red blood cells and other contaminants. To isolate ECs, both HUAs and the HUV were separately incubated in 3 ml of 1 mg/ml collagenase/dispase (C/D) solution (ROCHE, 10269638001) for 30 min at 37 °C. The C/D solution containing ECs was harvested, and vessels were washed with 3 ml PBS to harvest remaining detached cells. Each 6-ml cell suspension was diluted to 10–15 ml with PBS and centrifuged 5 min at 110 g. Each pellet was resuspended in Endothelial Cell Medium 2 (PromoCell, C-22011) and transferred to a 25-cm^2^ flask. To promote cell growth and development, the medium was supplemented with 10% heat-inactivated FBS (FBS Supreme, PAN-Biotech, P30-3031) until passage two (P2), after which FBS was removed due to its potential impact on sexual dimorphism of the cells [9] and VSMCs phenotype [10, 11].

#### VSMC isolation and culture

Vessels used for EC isolation were washed in PBS to discard remaining isolated ECs, cut into approximately 1-mm^2^ pieces and transferred to a 24-well plate (8–10 pieces per well; 6 wells per vessel type). Pieces of both HUAs were evenly distributed together in 6 wells, while HUV explants were placed in a further 6 wells. To facilitate explant adsorption to the bottom of the wells, pieces were allowed to adhere but not to over-dry for approximatively 5–10 min at room temperature (RT°) before gently adding only 150 µl of medium M231 (Human Vascular Smooth Muscle Cell Basal Medium M231, Gibco, M231500) supplemented with Smooth Muscle Growth Supplement (SMGS, Gibco, S00725) and 20% heat-inactivated FBS. The next day, the culture medium volume was completed to 500 µl. Tissues were removed if they detached or after 4–10 days. Visual inspection during the first days helped exclude contaminated wells or wells containing cells with EC phenotype. Typically, 2–6 visually optimal wells were pooled together and further processed. Cells were trypsinized and subcultured in 25- or 75-cm^2^ flasks depending on the quantity of harvested VSMCs. From P2, FBS was removed from the medium. VSMCs were subcultured when reaching 80–95% confluence or before over-confluence symptoms occurred in the densest areas.

### Characterization

#### Morphology

All cell types were visually inspected during early development and identified by their typical phenotype under phase contrast microscope. ECFCs and ECs have a cobblestone shape; VSMCs exhibit a spindle shape and a “hill-and-valley” pattern. In addition, ECFCs appear as colonies.

#### Western blot (WB)

Cell pellets for WB were obtained by trypsinization of three 75-cm^2^ flasks at early passages (P2-P3) followed by a wash with PBS and a 5-min centrifugation at 110 g to discard any medium traces and stored at -80 °C until protein extraction. Pellets were resuspended in 450 µl lysis buffer {50 mM HEPES, 1 mM EDTA, 1 mM EGTA, 10% glycerol, 1 mM DTT, 5 μg/ml pepstatin, 3 μg/ml aprotinin, 10 μg/ml leupeptin, 0.1 mM 4-(2-aminoethyl)benzenesulfonyl fluoride hydrochloride (AEBSF), 1 mM sodium vanadate, 50 mM sodium fluoride, and 20 mM 3-[(3cholamidopropyl)dimethylammonio]-1-propanesulfonate (CHAPS)} and went through 3 freeze/thaw cycles in liquid nitrogen and 37 °C water bath. Lysates were centrifuged at 3000 g for 10 min at 4 °C; supernatant protein concentration was quantified using a BCA protein assay kit (Pierce, catalog number 23227) according to the manufacturer’s instructions. WB was performed as previously described [1] using 60 µg of proteins per lane. The primary antibodies were directed against ERG (1:1000, Abcam, ab92513), endothelial nitric oxide synthase (eNOS) (1:200, Becton Dickinson, 610296), calponin 1 (Calp1) (1:5000, Abcam, ab46794), alpha-smooth muscle actin (SMA) (1:250, Sigma, A2547) and von Willebrand factor (VWF) (1:500, Cloud-Clone, PAA833Hu01). The secondary antibodies were IRDye 800 Donkey anti-mouse and IRDye 680 Donkey anti-rabbit (1:10,000, LI-COR Biosciences, 926-32212 and 926-68073). Visualization was done using an Odyssey Infrared Imaging System (LI-COR) and brightness was adjusted using ImageJ software.

As neither ECs nor VSMCs have a fully dedicated marker, preliminary experiments were conducted to determine, based on the literature, which proteins would be most suitable as biomarkers for differentiating ECs from SMCs in this project (data not shown). Using WB, we thus selected proteins found in both native HUV and HUAs from male and female newborns, but detected only in endothelial-like cells (ECs and ECFCs) or SMCs, in order to allow exclusion of cross-contamination between ECs and SMCs in cell cultures derived from umbilical vessels.

#### Immunocytofluorescence (ICF)

12,000 cells (P3-P4) per well were plated on a polymer 8-Chambers slide (μ-slide 8-well IbiTreat, Ibidi, 80826-IBI) coated with gelatin and were allowed to adhere overnight at 37 °C in a humidified and controlled atmosphere containing 5% CO_2_. Cells were then fixed according to the targeted protein as follows: cold methanol 1 min on ice for SMA and VWF; cold acetone 10 min on ice for eNOS and Calp1; paraformaldehyde 4% 10 min RT° for ERG. Additional wells with the same fixation conditions were used for unstained controls (without primary antibodies).

After fixation, slides were dried out and kept frozen at − 20 °C until ECs and VSMCs were ready to be processed simultaneously. Following a 5-min permeabilization with Triton 0.25% at RT°, cells were blocked 20 min at RT° with goat serum 4%, incubated overnight at 4 °C with the same primary antibodies as for WB diluted in blocking solution (1:100), washed, and incubated with goat anti-rabbit Cy2 (1:1000, Abcam, ab6940) and anti-mouse Alexa 568 (1:100, Life Technologies A-11004) for 1 h at RT°. Finally, cells were washed and covered using a non-hardening mounting medium containing DAPI. Images were taken with a Zeiss inverted microscope using a 20 × objective and optimized for visualization using ImageJ.

#### Polychromatic flow cytometry (PFC)

ECFC profile was confirmed by PFC using conjugated antibodies against CD31 (PE, BioLegend, 303106), CD146 (APC, BioLegend, 361016) and CD45 (BV 421, BioLegend, 368522) according to [5] and manufacturer’s instructions. Briefly, 300,000 cells (P2-P5) were split into 3 tubes: one with all three antibodies (total staining), one unstained control without antibodies, and one for viability test (Zombie NIR™ Fixable Viability Kit, BioLegend, 423105). Cells were incubated 20 min with the viability dye 1:500 at RT°, or with primary antibodies 1:40 on ice. After one wash for the viability test or two washes for the stained and unstained tubes, flow cytometry was performed on a Cytoflex S (V4-B2-Y4_r3, C09766) using CytExpert software (v2.3.1.22). Approximately 20,000 events were recorded in the FSC/SSC gated population during each read. The lasers (L) and filters (F) specifications of the Cytoflex S were as follows: PB450 (CD45) L:405 F:450/45, PE (CD31) L:561 F:585/42, APC (CD146) L:638 F:660/10, APC-A750 (Zombie NIR™) L:638 F:780/60.

Analysis was performed using the free web-based interface available at https://floreada.io/. First, cell population was gated using forward/side scatter (FSC/SSC) to exclude cellular debris; cell death was subsequently controlled to be less than 1% of the gated population with viability dye; finally, marker expression was measured on single parameter histogram and two-parameters density plot, with positive threshold based on unstained controls. No spectral overlap (Fig. S3) nor non-specific signal were detected, except for the viability dye which was run separately, so no compensation mechanisms were necessary. PFC experiment has been designed and optimized as detailed in Supplementary Information (Online Resource 1).

## Results

The profile of the biological samples used in this report and cell culture outcome for each sample are presented in Table S1. The success rate for each vascular cell type and the time required between cell isolation and first passage are summarized in Table 1.

**Table 1:** Success rate for each vascular cell type and time from cell isolation to subculture

	Females	Males	Total
ECFCs			
Blood samples processed	6	7	13
Successful cell culture	4	5	9
Successful characterization	4	5	9
ECFC success rate (%)	66.7	71.4	**69.2**
Days until P1 (median [range])	25 [16–30]	26 [25–34]	**26 [16–34]**
HUAECs			
Samples HUA processed	6	8	14
Successful cell culture	6	8	14
Successful characterization	5	7	12
HUAEC success rate (%)	83.3	87.5	**85.7**
Days until P1 (median [range])	15 [8–24]	11 [6–15]	**11.5 [6–24]**
HUVECs			
Samples HUV processed	6	8	14
Successful cell culture	6	8	14
Successful characterization	5	8	13
HUVEC success rate (%)	83.3	100	**92.9**
Days until P1 (median [range])	17 [15–26]	15 [9–21]	**15 [9–26]**
HUASMCs			
Samples HUA processed	6	8	14
Successful cell culture	6	8	14
Successful characterization	5	8	13
HUASMC success rate (%)	83.3	100	**92.9**
Days until P1 (median [range])	15 [13–20]	13.5 [11–15]	14 [11–20]*
HUVSMCs			
Samples HUV processed	6	8	14
Successful cell culture	5	8	13
Successful characterization	3	6	9
HUVSMC success rate (%)	50	75	**64.3**
Days until P1 (median [range])	17 [16–22]	16.5 [15–20]	17 [15–22]*

Success rate for each cell type is based on two criteria: (1) successful cell culture (successful isolation of cells that proliferate to subculture) and (2) successful characterization (presence of the appropriate biomarkers). No statistically significant difference was found between the success rates of the different cell types using a Chi-square test. Days until P1 correspond to the number of days from cell isolation to first passage (P1)

*Significant difference between HUVSMCs and HUASMCs (p = 0.0156, Wilcoxon matched-pairs signed rank test); no significant difference was found between days until P1 for HUAECs and HUVECs (p = 0.0801)

### Vascular cell isolation and culture

#### ECFCs

**Fig. 2:**
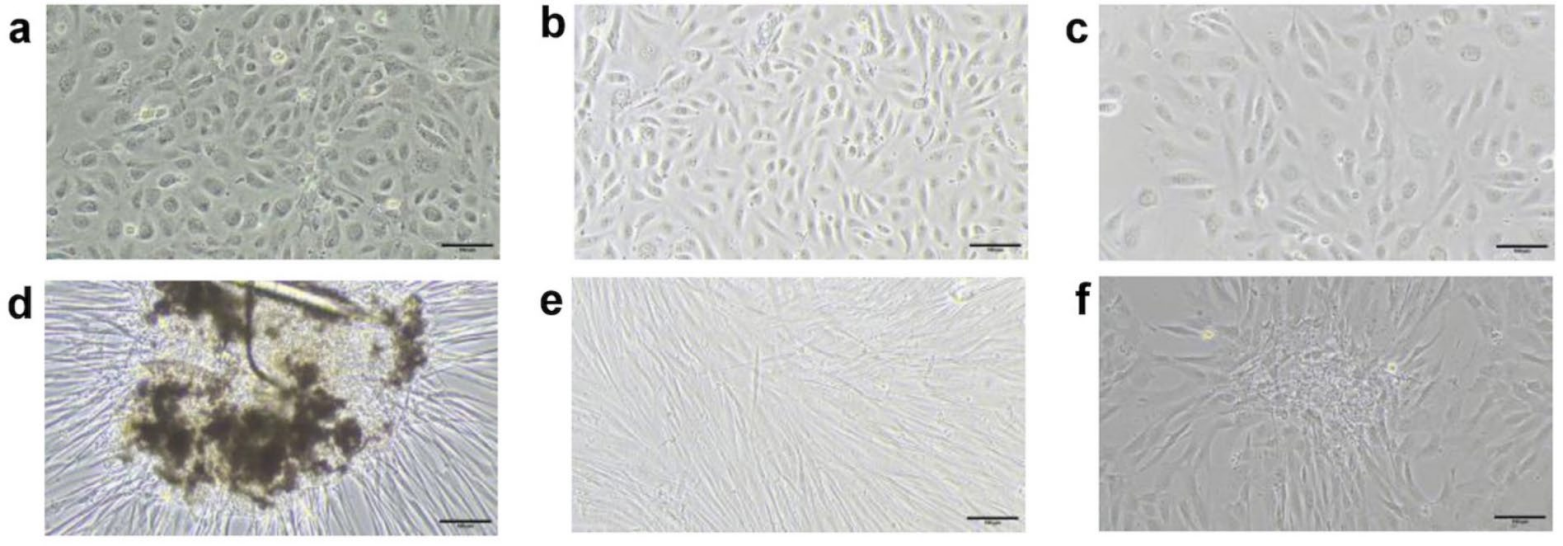
Brightfield images of ECFCs (**a**), HUAECs (**b**) and HUVECs (**c**) with their typical cobblestone phenotype. HUASMCs expanding from an explant after few days (**d**) before starting to form a “hill-and-valley” pattern (**e**). HUVSMCs and their less elongated phenotype, forming a nodule (**f**). Images were taken with a 10 × objective, scale bar = 100 µm

The first colonies appeared after 10–30 days with a tightly packed cobblestone phenotype (Fig. 2a). We noticed that cell size varied with density and proliferation rate, but this did not affect characterization outcomes. ECFCs were subcultured approximately 26 days after isolation (Table 1).

ECFC culture had a success rate of 9/13 (69%) (Table 1). All successful ECFC cultures met the characterization criteria using PFC and WB.

#### ECs

HUAECs (Fig. 2b) and HUVECs (Fig. 2c) were the easiest cell types to isolate and cultivate. Both exhibited a classic cobblestone phenotype and high proliferation rates. No morphological distinction was observed between HUAECs and HUVECs. As for ECFCs, we sometimes observed increased cell size when confluency and proliferation rates were lower than expected, but this did not affect characterization outcomes. HUAECs often provided better yields than HUVECs and were the first to be subcultured after approximately 11 days, compared to 15 days for HUVECs (Table 1). No significant difference was found in the number of days between isolation and the first subculture (P1) for HUAECs and HUVECs (p = 0.0801, Wilcoxon matched-pairs signed rank test).

1/14 HUAEC culture was contaminated by VSMCs, which was easily detected by brightfield microscopy due to the distinct elongated shape of VSMCs compared to ECs, and was later confirmed by WB. Another one failed to meet the characterization criteria, and 1/14 HUVECs did not show ERG expression by WB, resulting in a success rate of 12/14 (86%) for HUAECs and 13/14 (93%) for HUVECs (Table 1).

#### SMCs

HUASMCs and HUVSMCs required the most rigorous attention, mainly during the explant phase where it was crucial to exclude any wells that might contain ECs. HUASMCs began migrating from explants after about 1 week (Fig. 2d), and further grew as spindle-shaped cells with “hill-and-valley” pattern (Fig. 2e). HUVSMC migration was often more spread around and beneath the explant tissue. HUVSMCs appeared less elongated but were able to form nodules [12] (Fig. 2f) and a “hill-and-valley” pattern.

For each umbilical cord, HUV and HUA explants were distributed in 4–6 wells for each vessel type to isolate VSMCs. Only visually satisfying wells were further processed and subcultured. The proportion of wells selected for subculture was significantly lower for HUVSMCs (43/80, 54%) than HUASMCs (74/80, 93%) (p = 0.0029, Wilcoxon matched-paired signed rank test) (Table S1). The number of days between explant culture and first passage was significantly greater for HUVSMCs (approximately 17 days) than HUASMCs (approximately 14 days) (Table 1).

Globally, 1/14 HUVSMC culture failed to start, while 1/14 HUASMC and 4/14 HUVSMC cultures were found, after characterization by WB, contaminated by ECs despite careful visual inspection and selection of the most promising wells. Therefore, the resulting success rates were 13/14 (93%) for HUASMCs and 9/14 (64%) for HUVSMCs (Table 1).

No bacterial or fungal contamination was observed in any cultures after Primocin® was introduced in our protocols.

### Characterization

**Fig. 3:**
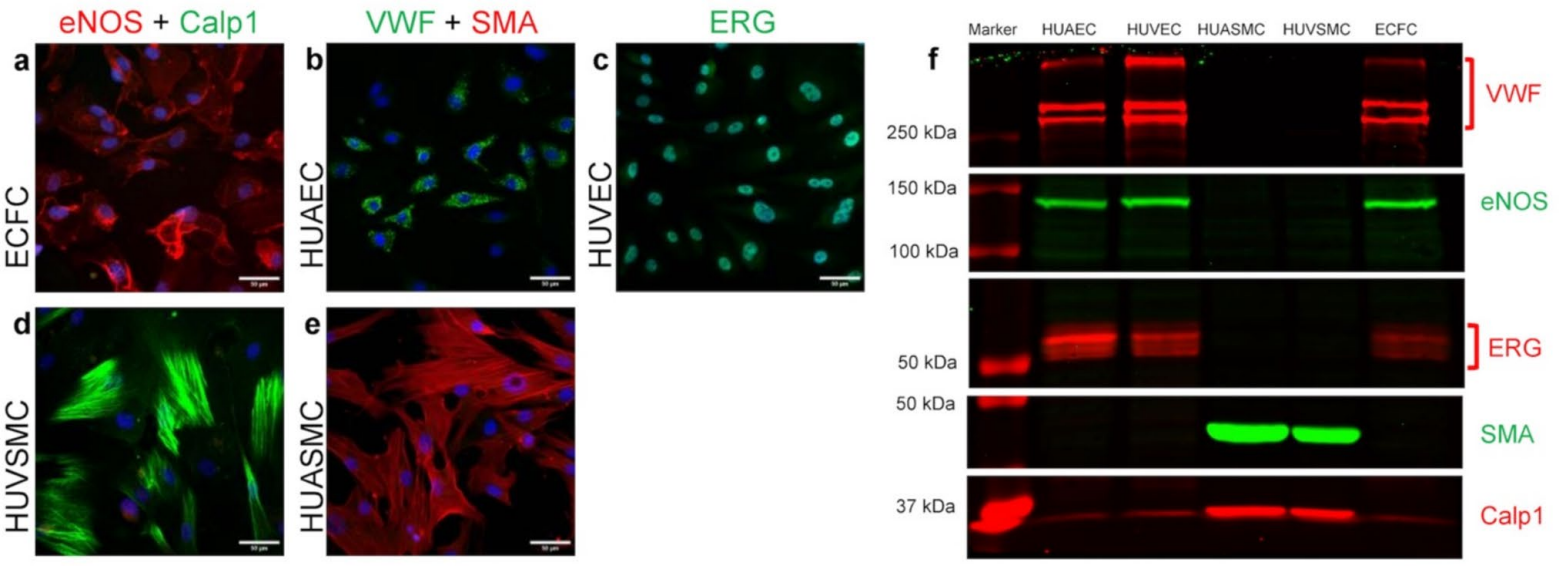
Representative results of immunocytofluorescence (**a**–**e**) and Western blot (**f**) used to characterize cell cultures. ECFCs (**a**), HUAECs (**b**), and HUVECs (**c**) are positive to eNOS (**a**), VWF (**b**), and ERG (**c**), but negative to Calp1 (**a**) and SMA (**b**). HUVSMCs (**d**) and HUASMCs (**e**) are positive to Calp1 (**d**) and SMA (**e**), but negative to eNOS (**d**), VWF (**e**) and ERG (Fig. S2). Nuclei staining with DAPI in blue. 20 × objective, scale bar = 50 µm. Western blot (f) confirms immunocytofluorescence data

Cell culture characterization was confirmed by WB and ICF (Fig. 3, S1-S2). Successful EC and ECFC cultures were defined by the presence of eNOS, VWF and ERG, and absence of SMA and Calp1; successful VSMC cultures showed the opposite profile (Fig. 3).

A slight band below the Calp1 staining was attributed to a non-specific signal from the anti-ERG antibody previously used on the same membrane (Fig. 3f, S1).

**Fig. 4:**
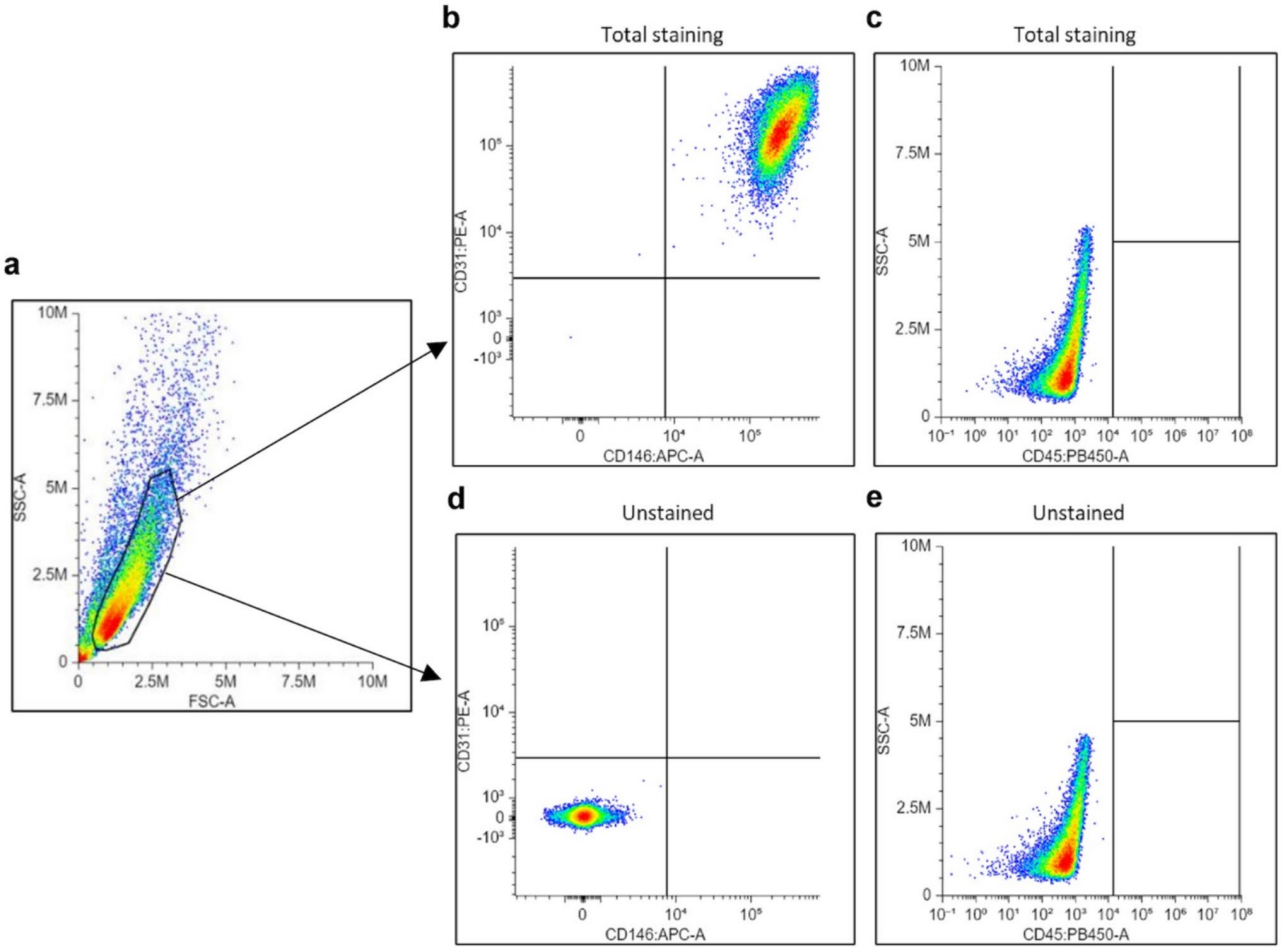
Representative Flow cytometry analysis and gating strategy of an ECFC sample. First panel (**a**) shows the ECFCs gating on an FSC/SSC density plot. The ECFC population was then immunophenotyped with CD31, CD146 and CD45 (**b**, **c**) according to the unstained sample (**d**, **e**). CD31 (PE) and CD146 (APC) are represented on a bivariant density plot (**b**, **d**) while CD45 (PB450) is represented alone (**c**, **e**). A total of approximately 34,000 events were recorded for each sample, more than 18,000 of which were gated as ECFC population. These graphs were obtained using Floreada.io as described in the Methods section

ECFCs isolated from 5 umbilical cord blood samples were characterized by PFC. Immunophenotyping revealed that over 99.9% of the cells were CD31^+^/CD146^+^, and 100% were CD45^–^ (Fig. 4, Table S1). Detailed validation controls are presented in the Supplementary Information (Online Resource 1).

## Discussion

This study aimed to develop a simple, rapid and cost-effective method for simultaneous isolation and culture of ECFCs, HUVECs, HUAECs, HUVSMCs and HUASMCs from the umbilical cord of a single patient.

First, cord blood is processed to harvest mononuclear cells using a Ficoll gradient, before plating and culture until obtaining ECFCs. Then, HUV and HUAs are carefully dissected, longitudinally opened to expose the lumen and incubated with collagenase/dispase to recover ECs. Finally, the vessels used to isolate ECs are washed and cut into small pieces to obtain VSMCs by migration from vascular explants. All cell types are then characterized by visual inspection, WB and ICF. ECFCs characterization is also confirmed by PFC analysis.

Numerous publications describe how to isolate ECs or VSMCs from human umbilical cords. On the basis of several previous reports, notably that by Martin de Llano [13], we addressed various issues and optimized the procedure to reduce costs, contamination risks, and technical complexity, allowing an easy application for laboratories without requiring any specialized equipment or knowledge.

The main step to simplify the procedure is to longitudinally open the vessels before enzymatic digestion to recover ECs, and to use explants for VSMCs. There is no need for a catheter, therefore reducing costs and handling, improving sterility and solving some issues like blood clots or enzymatic solution leakage.

It is worth noting that Primocin® solved one of the major problems we faced during protocol optimization by completely preventing any contamination. Moreover, its anti-mycoplasma effect could be a valuable help given the common presence of genital mycoplasmas [14], their in-vitro persistence, and their potential impact on various experiments.

ECs were the easiest cells to isolate and cultivate, achieving a success rate of about 90%. Contamination by VSMCs is unlikely to persist as these cells seem to require different supplements for proper growth and will either go through apoptosis or be outgrown by ECs.

ECFC culture had a success rate of about 70%, consistent with previous reports indicating a failure to isolate ECFCs from about 25–30% of healthy donors [15].

VSMCs were the cells requiring most attention, with higher success rate for HUASMCs (93%) compared to HUVSMCs (64%). This could be linked to the thinner muscular wall of HUV and the weaker explant attachment to the well bottoms compared to HUA. Moreover, differentiating HUVSMCs from ECs can be challenging due to their similar appearance, particularly before they form a distinct “hill-and-valley” pattern. It has already been described that subpopulations of VSMCs can even have a cobblestone appearance [16, 17]. However, as the proportion of visually satisfying wells selected for subculture was lower for HUVSMCs (55%) than HUASMCs (93%), increasing the initial number of wells containing HUV explants could improve the success rate for HUVSMCs. Based on our observations, twice as many wells should be prepared with explants of HUV as of HUA.

Given the risk of cross-contamination, distributing vascular explants in several small wells (instead of putting them into a larger culture dish) helps to limit the risk of having an entire SMC culture contaminated with ECs, as wells that visually appear suspicious can be eliminated, so that only the most promising wells will be processed and subcultured.

Cell culture characterization by WB, ICF and PFC yielded concordant outcomes, confirming the reliability of this protocol. Due to the various phenotypes and subsequent protein expression changes reported in the literature [10–12, 18–20], neither ECs nor VSMCs have a fully dedicated marker. It is therefore recommended to combine multiple markers [12, 19], selecting proteins unique to ECs or VSMCs to exclude cross-contaminations. Consequently, we selected several proteins that we found in both native umbilical vessels, but detected only in ECs (eNOS, VWF and ERG) or SMCs (SMA and Calp1), to allow detection of cross-contaminations. Other studies classically used SMA, smooth muscle-myosin heavy chain, Calp1, SM22-alpha or h-caldesmon for SMCs and CD31 or VWF for ECs [10, 12, 13, 18–20].

It would be interesting to investigate the expression of some molecules used to distinguish between cells isolated from arteries and veins, although this step is not necessary in the proposed procedure since we know from which vessel the cells derived. There is no fully dedicated marker for either vessel. Among those classically used, Eph B4 receptor and its ligand ephrin-B2 have been described as preferentially expressed in veins and arteries, respectively [21–23]. However, performing preliminary experiments to determine if they allow discrimination between cells isolated from HUV and HUAs in our project showed sex differences in native vessels: ephrin-B2 was significantly increased in HUA compared to HUV in males, but not in females (Online resource 2). These observations highlight the need to consider biological sex when establishing biomarkers. Moreover, differential expression of these arteriovenous biomarkers was found to vary between adult and umbilical vessels [24]. As there is increasing evidence that Eph/ephrin family play key roles in cardiovascular development and disorders [25, 26], the relative expression of these proteins in umbilical vessels from males and females will be investigated in both appropriate for gestational age (AGA) and growth-restricted newborns.

Regarding the time required between cell isolation and first passage, it was similar for HUAECs and HUVECs, but longer for HUVSMCs than HUASMCs. Other comparisons are not relevant, as the quantity of cells initially cultured was not standardized. Cell proliferation will be studied later to further characterize umbilical vascular cells isolated from AGA and IUGR male and female newborns.

Indeed, the development of this methodology for isolating vascular cells from umbilical cords is a steppingstone in our ongoing study of altered regulation of the human umbilical circulation in IUGR and the influence of fetal sex. In particular, we will compare, for each cell type, functional and molecular properties (including genetic profile) in cells isolated from AGA and IUGR male and female newborns. We will also compare, within each study group, HUVSMCs with HUASMCs, HUAECs with HUVECs, as well as ECFCs with differentiated HUAECs and HUVECs.

In conclusion, this protocol provides a reliable and effective method for simultaneous isolation and culture of ECFCs, ECs and VSMCs from cord blood, HUV and HUAs collected from the same patient.

This will enable optimal use of each biological sample and direct comparison between different cell types. This approach could facilitate the creation of a biobank containing cryopreserved ECFCs, HUVECs, HUAECs, HUVSMCs, HUAECs, as well as native HUV and HUAs, linked to clinical data, offering further research opportunities. Isolation and culture of vascular cells from umbilical cords of healthy or sick neonates would lead to a better understanding of human umbilical circulation under physiological or pathological conditions. In addition, considering the sex of the newborns from whom these cells originate will allow to assess the influence of biological sex, which should be considered a key factor in cardiovascular research and clinical management. This will contribute to the development of targeted therapeutic strategies in the future.

## Supplementary Information

Below is the link to the electronic supplementary material.

Supplementary file1 (PDF 5940 KB)

## Data Availability

Data are provided within the manuscript and supplementary information files.
