# Supplementary material for: Simultaneous isolation and culture of endothelial colony-forming cells, endothelial cells and vascular smooth muscle cells from human umbilical cords
Source: Mol Biol Rep. 2025 Mar 13;52(1):302. doi: 10.1007/s11033-025-10418-1 (PMC11906538; doi:10.1007/s11033-025-10418-1)
Supplement: Supplementary file 1 — Supplementary file1 (PDF 5940 KB) [file 11033_2025_10418_MOESM1_ESM.pdf]

Simultaneous isolation and culture of endothelial colony-forming cells, endothelial cells and vascular smooth muscle cells from human umbilical cords

Marie-Lotus Burger[1], Steeve Menétrey[1], Catherine Ponti[1], Karine Lepigeon[2], Joanna Sichitiu[3], Anne-Christine Peyter[1*]

[1] Neonatal Research Laboratory, Department Woman-Mother-Child, Lausanne University Hospital and University of Lausanne, Lausanne, Switzerland

[2] Clinic of Gynecology and Obstetrics, Department Woman-Mother-Child, Lausanne University Hospital and University of Lausanne, Lausanne, Switzerland

[3] Ultrasound and Fetal Medicine Unit, Department Woman-Mother-Child, Lausanne University Hospital and University of Lausanne, Lausanne, Switzerland

* Corresponding author: Anne-Christine.Peyter@chuv.ch

SUPPLEMENTARY INFORMATION

Figure S1

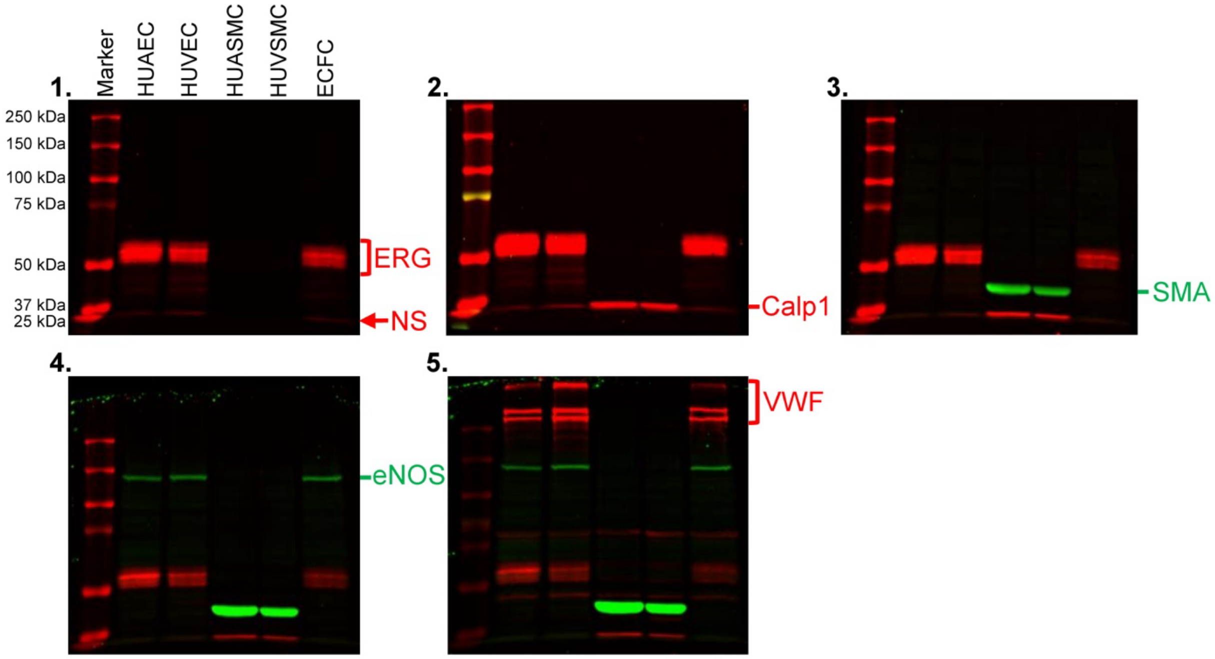

Fig. S1 Representative Western blot of the cell culture characterization process with successive antibody staining: (1) ERG, (2) Calp1, (3) SMA, (4) eNOS, (5) VWF. The red arrow in the first panel shows the slight bands considered as non-specific (NS) that need to be distinguished from the following Calp1 staining. The antibodies used in WB for the characterization of cell cultures were successively run on the same membrane. A picture was taken after each incubation to document the superposition of the antibodies, which was optimized according to the non-specific signals that can occur.

Figure S2

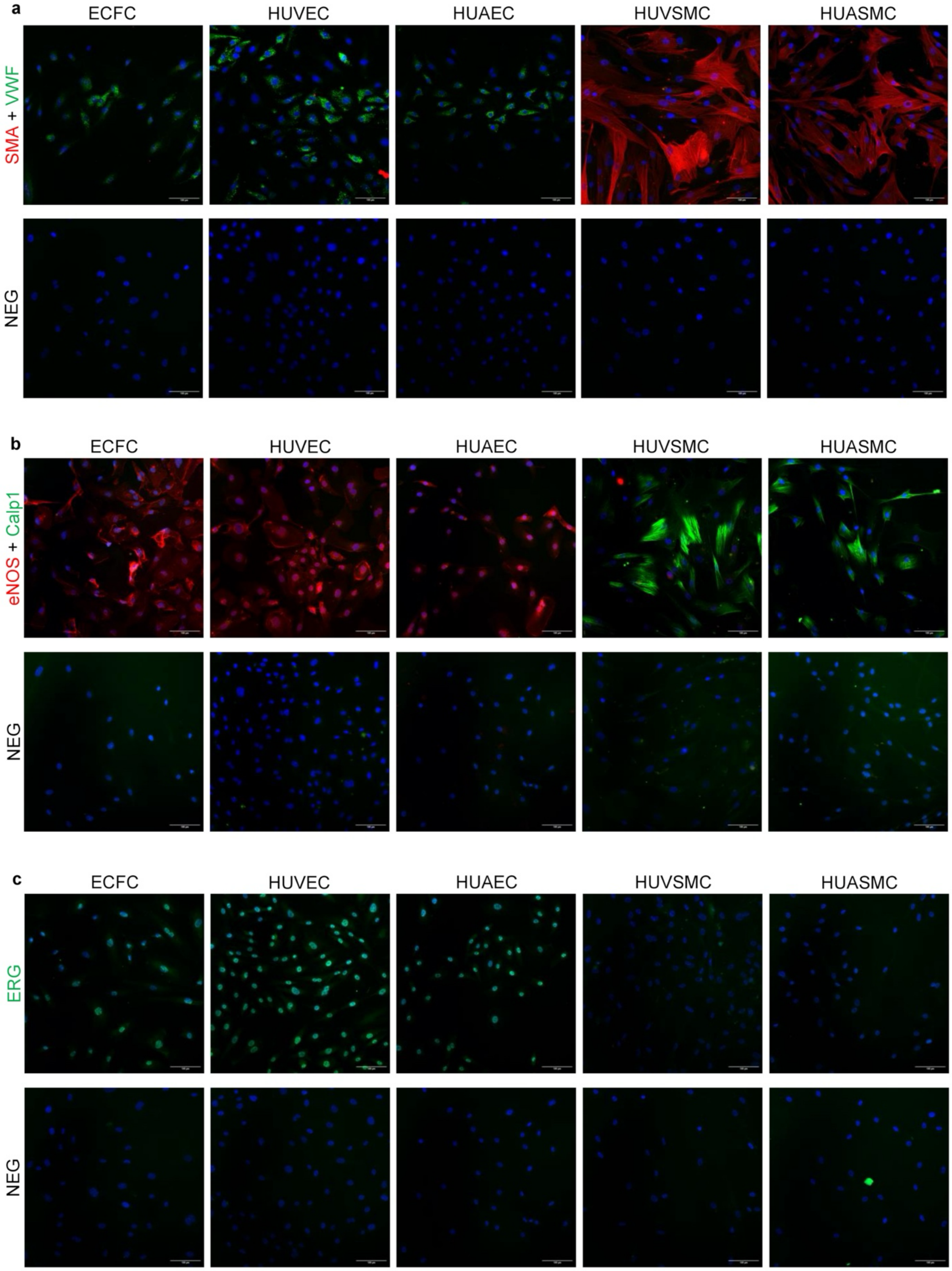

a
ECFC
HUVEC
HUAEC
HUVSMC
HUASMC
SMA + VWF
NEG
b
ECFC
HUVEC
HUAEC
HUVSMC
HUASMC
eNOS + Calp1
NEG
c
ECFC
HUVEC
HUAEC
HUVSMC
HUASMC
ERG
NEG

Fig. S2 Representative illustration of a complete ICF experiment. Each panel represents all cell types with VWF (green) and SMA (red) staining (a), Calp1 (green) and eNOS (red) staining (b), and ERG (green) staining (c), and their respective negative control (NEG). DAPI is in blue. Images were taken with a 20x objective, scale bar = 100µm. ICF staining was run at the same time on each cell type. A negative control without primary antibody was assessed for each condition. The contrast settings applied here were optimized for the visualization of each antibody but was strictly the same for the stained and unstained sample (NEG) of each cell types. VWF and SMA, and eNOS and Calp1 were incubated together, while ERG was run alone. Although the level of staining was not the same across all cells and sometimes was not detected at all in some of them, nothing was suggesting a wrong cell type. Therefore, we assumed it was simply relying on variable expression amongst the cells as already reported [10].

Table S1 Profile of the biological samples used in this report and cell culture success

Neonatal data								Cell culture success (successful culture / successful characterization)					Satisfying wells/total wells with explants	
Sex	Gestational age (weeks)	Birth weight (g)	Length (cm)	Head circumference (cm)	Cord diameter (cm)	Maternal age (years)	Cord blood volume (ml)	ECFCs	HUAECs	HUVECs	HUASMCs	HUVSMCs	HUASMCs	HUVSMCs
M	41.6	3220	49	34.0	9.43	24.9	2.0	-	+/+	+/+	+/+	+/+	4/4	4/4
M	41.6	3240	51	36.0	10.57	36.9	∅	∅	+/+	+/+	+/+	+/+	4/4	2/4
M	41.3	3260	49	34.0	9.24	32.1	5.4	+/+	+/+	+/+	+/+	+/-	6/6	2/6
M	41.1	3750	51	35.0	12.52	30.7	1.4	-	+/+	+/+	+/+	+/-	6/6	2/6
M	41.3	3695	50	36.0	8.22	37.9	5.0	+/+	+/+	+/+	+/+	+/+	6/6	3/6
M	42.3	3520	50	33.5	11.00	26.5	7.0	+/+	+/+	+/+	+/+	+/+	6/6	6/6
M	41.9	4130	51	36.5	9.28	36.0	2.3	+/+	+/-	+/+	+/+	+/+	6/6	1/6
M	41.4	4050	53	36.0	8.71	37.7	3.4	+/+	+/+	+/+	+/+	+/+	6/6	5/6
F	41.7	3000	47	33.0	10.08	29.3	2.8	-	+/+	+/+	+/-	+/+	4/6	2/6
F	40.3	2500	50	33.0	8.55	30.5	4.0	+/+	+/-	+/+	+/+	+/-	6/6	4/6
F	42.0	4090	51	36.0	9.83	28.6	2.8	+/+	+/+	+/+	+/+	+/+	6/6	6/6
F	39.0	2790	47	33.0	9.55	30.4	2.9	+/+	+/+	+/+	+/+	+/+	6/6	3/6
F	42.0	3930	52	34.5	8.86	29.7	5.5	-	+/+	+/-	+/+	+/-	2/6	3/6
F	39.3	2475	45	32.0	8.92	42.0	5.5	+/+	+/+	+/+	+/+	-	6/6	0/6

M, male; F, female. The cord blood volume corresponds to the volume collected in the umbilical vein to isolate ECFCs. Cell culture success is based on two criteria: (1) successful culture (successful isolation of cells that proliferate to subculture) and (2) successful characterization (presence of the appropriate biomarkers) (+ yes, - no). ∅ ECFCs were not isolated because the cord blood was collected more than 12 hours earlier. For SMCs, explants from each vessel were distributed in 4-6 wells; the number of visually satisfying wells selected for subculture was reported to the number of wells prepared with explants. This global proportion of promising wells was significantly lower for HUVSMCs (43/80, 54%) than HUASMCs (74/80, 93%) (p=0.0029, Wilcoxon matched-pairs signed rank test).

Online Resource 1

PFC immunostaining validation procedure

To design an efficient flow cytometry experiment, we first checked if there was no spectral overlap of the antibodies staining by running each antibody alone versus an unstained control (without antibodies). Using single parameter histograms, we were able to exclude any spectral overlap with our antibodies (Fig S3). However, we observed that CD146 (APC) was spilling over into the Zombie NIR™ Viability detector APC-A750. As we were observing very high level of staining with CD146 and a very low staining with the viability dye, we chose to use the viability marker in a separate assay instead of using compensation mechanisms.

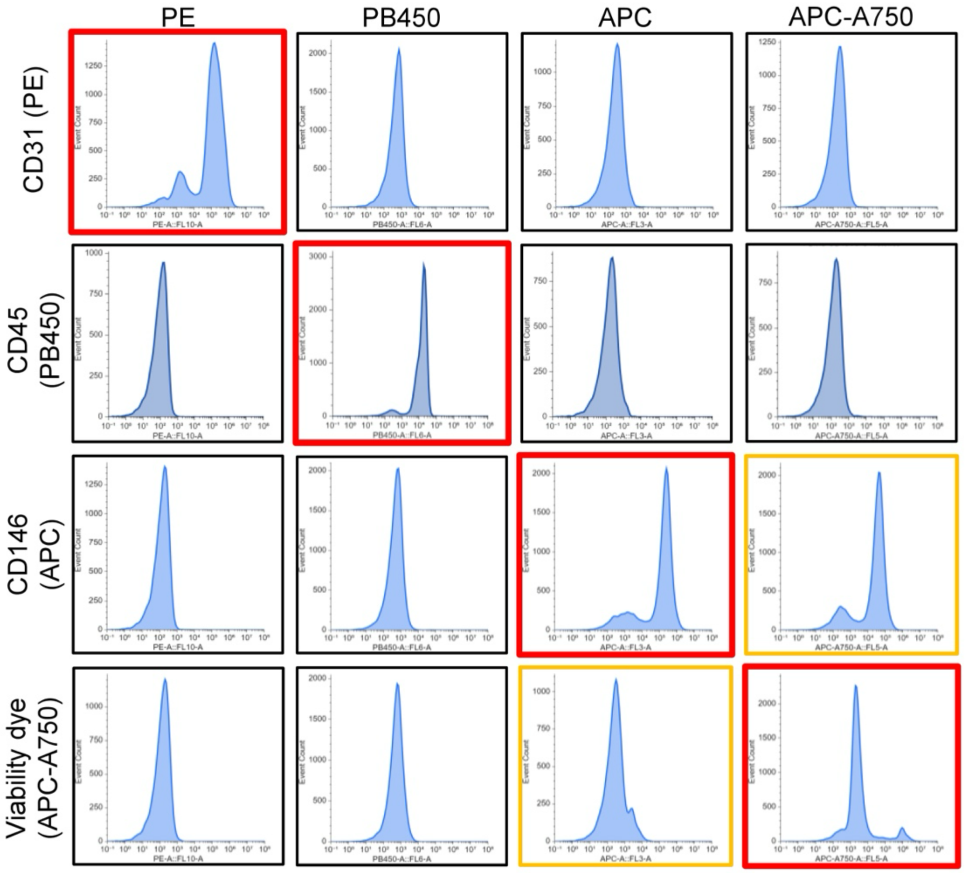

Fig. S3 Single parameters histograms with single staining of ECFCs (except for CD45 where PBMCs are shown instead) represented line by line. The columns represent each spectrum used during these experiments. Red boarders indicate the filters corresponding to the fluorochrome. The orange boarders indicate the spectral overlapping. A total of approximately 30’000 events were recorded for each sample. These graphs were obtained using Floreada.io as described in the methods section.

To check the efficacy of the viability dye and the FSC/SSC gating strategy, we induced partial cell death by heating cells at 50°C for 15 min before the staining steps. This method was chosen because it does not require any chemical substances, therefore avoiding any interference with the detection steps. When compared to the not-heated cells, we clearly observe an increase in the population of presumably dead cells (Fig. S4a-b) rising from 5.4% to 30.2% of all the events.

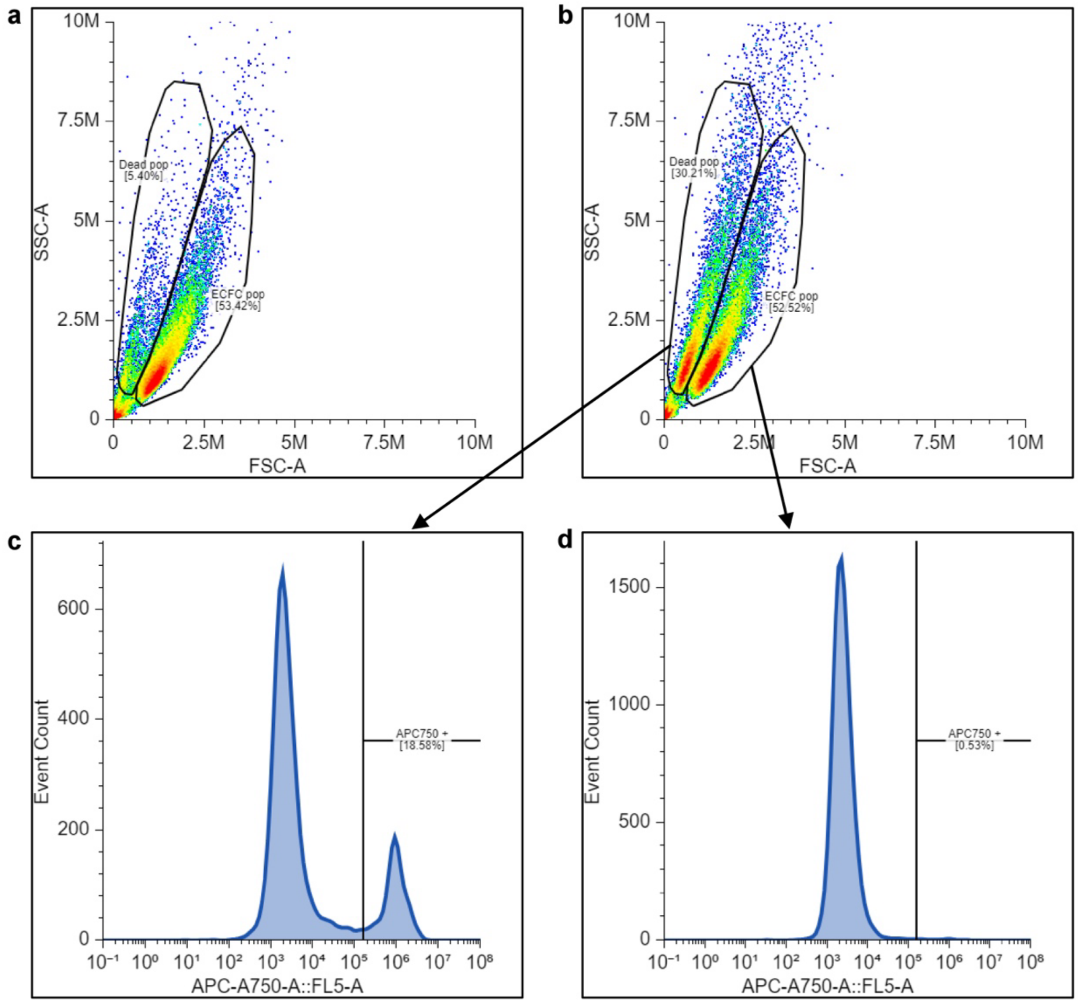

Fig S4 Forward and side scatter (FSC/SSC) density plot and the gating strategy of ECFCs sample (a) and heated ECFCs sample (b) to induce cell death. Panels c-d show single parameter histograms of the viability dye (APC-A750) from the gated population of dead cells (c) and ECFCs (d). In both normal sample and heated sample, the gated population of intact cells represents approximately 53% of all events, while the population of presumably dead cells rise from 5 to 30% (a-b). A total of approximately 30’000 events were recorded for each sample. These graphs were obtained using Floreada.io as described in the methods section.

By measuring the level of staining of the viability dye in the two sub-populations of events, we noticed that only 18% of the population of dead cells was positive (Fig. 4c) to the dye, suggesting either the presence of a sub-population of alive ECFC, or the uncomplete staining of all the dead cells. However, we observed that not only the population of presumably dead cells also express the same staining profile as the ECFC population - $CD31^+$ (>95%), $CD45^-$ (<1%) and $CD146^+$ (>95%) (data not shown) - but also the ECFC population stained by the viability dye is less than 1% (Fig. S4d). It is worth noting that according to the manufacturer, this dye does not stain apoptotic cells. Taken together, these results suggest that the FSC/SSC gating strategy sufficiently represent the whole culture of ECFCs, and that the proportion of dead cells in this population is low enough to raise the eventuality of false-positive to be negligible.

As expected, we never obtained positive signal with the antibody against CD45 in our ECFCs. Therefore, we decided to confirm the specificity of this antibody using enriched lymphocytes from human Peripheral Blood Mononuclear Cells (PBMCs) that are known to express CD45 [27-28]. Indeed, we obtained more than 99% of CD45 positive cells, 49% of CD31 positive, and less than 1% of CD146 positive (Fig. S5 & Table S2). One HUVSMC and one HUAEC sample were also used as validation controls. HUVSMCs were 94% $CD146^+$, and less than 0.1% were $CD31^+$ or $CD45^+$ (Table S2). HUAECs showed the same characteristics than ECFCs with more than 99.5% $CD31^+/CD146^+/CD45^-$ (Table S2). These results confirm the reliability of the three antibodies used.

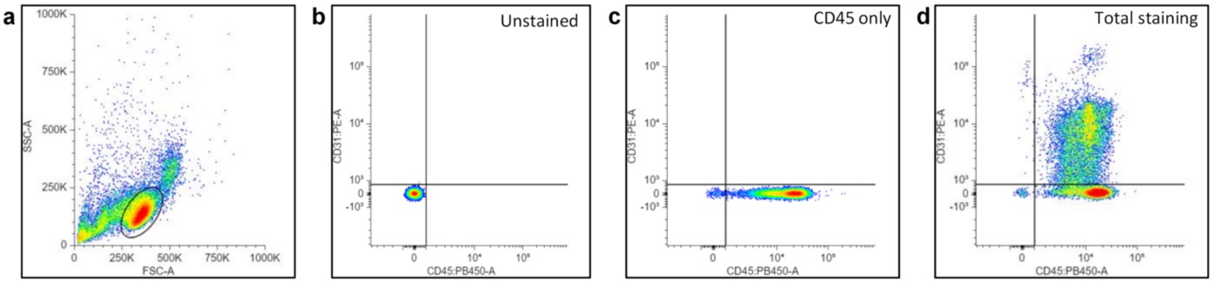

Fig. S5 Flow cytometry analysis and gating strategy of the PBMCs sample. First panel (a) shows the PBMCs gating on an FSC/SSC density plot. The second panel (b) shows the unstained sample for CD31 (PE) and CD45 (PB450) used to define the positive threshold. Third panel (c) shows the sample stained with CD45 only. Last panel (d) shows CD31 and CD45 repartition of the sample stained with CD31, CD146 (not shown) and CD45. A total of approximately 24’000 events were recorded for each sample, more than 17’000 of which were gated. These graphs were obtained using Floreada.io as described in the methods section.

	CD31+ (%)	CD146+ (%)	CD45+ (%)	CD31+ CD146+ (%)	Gated dead cells (%)	Total dead cells (%)
**ECFC stained**	**99.99**	**99.95**	**0.00**	**99.95**		
ECFC unstained	0.00	0.00	0.00	0.00	0.00	0.00
ECFC viability dye	0.00	0.00	0.00	0.00	0.52	2.23
**HUVSMC stained**	**0.02**	**94.33**	**0.02**	**0.05**		
HUVSMC unstained	0.01	0.01	0.01	0.00	0.00	0.00
HUVSMC viability dye	0.00	0.11	0.02	0.00	0.59	2.89
**HUAEC stained**	**99.80**	**99.89**	**0.14**	**99.63**		
HUAEC unstained	0.00	0.00	0.00	0.00	0.00	0.04
HUAEC viability dye	0.01	0.02	0.02	0.00	1.30	6.01
				**CD31+ CD45+ (%)**		
**PBMC stained**	**49.17**	**0.18**	**99.24**	**50.00**		
PBMC unstained	0.00	0.00	0.00	0.00		
PBMC CD45 only	0.00	0.00	98.99	0.00		
PBMC viability dye	0.00	0.01	0.00	0.00	0.06	5.09

Table S2 Relative results obtained by flow cytometry on ECFCs, HUVSMCs, HUAECs and PBMCs on the FSC/SSC gated population. All values are percent of the gated population, except for the last column on the right which shows the total staining of the viability dye on all events (except debris).

Online Resource 2

Ephrin-B2 in human umbilical vessels

The transmembrane ligand ephrin-B2 and its receptor Eph B4 are known to play a key role in cell sorting and segregation processes, notably between arterial and venous endothelial cells [26].
To investigate if ephrin-B2 could serve as a differential arteriovenous marker to further distinguish between cells isolated from umbilical arteries or veins, we first assessed its relative abundance in HUA and HUV homogenates by Western blot (Fig. S6).
Western blots were performed using umbilical vessels collected and processed in our previous study [2]. For each vessel type and experimental group, homogenates of HUV or HUA isolated from 40 patients (40 appropriate for gestational age (AGA) females and 40 AGA males) were randomly distributed into 4 pools per group. Briefly, samples containing 60µg of proteins were loaded on a 12% polyacrylamide gel, submitted to SDS-PAGE and transferred to a nitrocellulose membrane. After blocking with casein, membranes were incubated with anti-ephrin-B2 (1:1'000, NovusBio, NBP1-84830), followed by IRDye 680 Donkey anti-rabbit (1:10'000, LI-COR Biosciences, 926-68073). Visualization was done using an Odyssey Infrared Imaging System (LI-COR). The ephrin-B2 signal was quantified using ImageJ software and normalized to the corresponding Ponceau S total protein staining. To allow comparison between the different membranes and groups, the specific protein content measured in each pool was reported to the amount measured in a "standard" sample (prepared with equal quantities of 3 female HUV, 3 male HUV, 3 female HUA and 3 male HUA, isolated from patients not included in the other pools) added on each membrane as previously described [2].

Fig. S6 shows that ephrin-B2 is detected in both HUV and HUA. Its relative protein content is significantly higher in HUA than HUV in AGA males, as expected based on previous publications [21-23]. However, in females, no significant difference is observed between HUV and HUA. These preliminary results suggest that ephrin-B2 abundance may be differently modulated depending on the sex of the newborn.
These findings underscore the need to consider biological sex when searching for arteriovenous biomarkers. Moreover, it is likely that Eph/ephrin proteins may also be modulated by pathological conditions like IUGR, thus requiring further investigations.

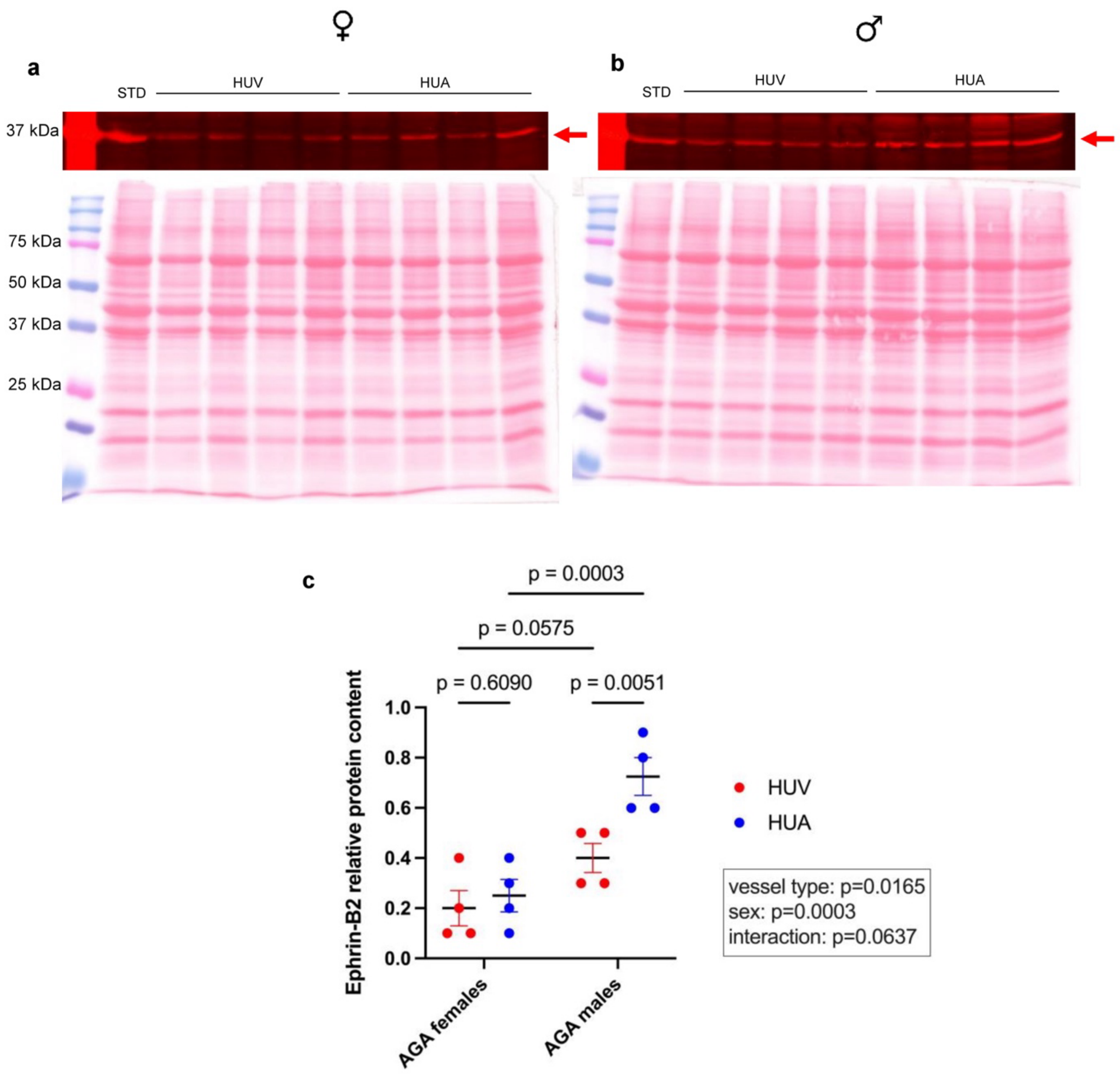

Fig. S6 Western blot analysis of ephrin-B2 in HUA and HUV homogenates from appropriate for gestational age (AGA) females (a) and males (b). The standard sample (STD) contains 3 female HUV, 3 male HUV, 3 female HUA and 3 male HUA. The other samples correspond to the different pools, each constituted of HUV or HUA isolated from 10 patients. The corresponding total protein staining with Ponceau S is shown below the anti-Ephrin B2 staining. For each pool, the target protein amount was normalized to the corresponding Ponceau S total protein staining and reported to the amount measured in STD. The resulting relative protein content is reported as individual values (c), with line at mean ± SE (n = 4 pools of 10 patients). Data were analyzed by two-way ANOVA (results are shown next to the graph) with uncorrected Fisher's LSD (P values obtained when comparing two groups are shown on the graph).

References

27. Zhang Y, Ma XZ, Zhao XY, Li JJ, Ma S, Pang ZD, Xu J, Du XJ, Deng XL, Wang JH. AGEs-RAGE-KCa3.1 pathway mediates palmitic acid-induced migration of PBMCs from patients with type 2 diabetes. Heliyon. 2023;9(4):e14823. doi: 10.1016/j.heliyon.2023.e14823.

28. Bellik L, Ledda F, Parenti A. Morphological and phenotypical characterization of human endothelial progenitor cells in an early stage of differentiation. FEBS Lett. 2005;579(12):2731-6. doi: 10.1016/j.febslet.2005.04.003.
